# Supplementation of Paraformic Acid as a Substitute for Antibiotics in the Diet Improves Growth Performance and Liver Health in Broiler Chickens

**DOI:** 10.3390/ani12202825

**Published:** 2022-10-18

**Authors:** Qinjin Wang, Jiaxing Niu, Yang Liu, Ning Jiao, Libo Huang, Shuzhen Jiang, Lei Yan, Weiren Yang, Yang Li

**Affiliations:** 1 Shandong Provincial Key Laboratory of Animal Biotechnology and Disease Control and Prevention, Department of Animal Science and Veterinary Medicine, Shandong Agricultural University, Daizong Street 61#, Tai’an 271018, China; 2 Shandong Wonong Agro-Tech Group Co., Ltd., Changning Street 118#, Weifang 261200, China; 3 Shandong New Hope Liuhe Group Co., Ltd., Jiudongshui Road 592-26#, Qingdao 266100, China

**Keywords:** antibiotic growth promoter, chicken, feed additives, formic acid, liver

## Abstract

**Simple Summary:**

Paraformic acid (PFA) is a hyperpolymer formed by polymerization of formic acid monomers, and PFA has a lower pungent smell and corrosiveness to the gastrointestinal tract compared with formic acid. The liver is a crucial metabolic organ, playing a momentous role in nutrients biosynthesis and metabolism, defense against bacterial invasion, and endotoxin clearance. However, in the condition of intensive poultry husbandry, the broiler’s liver is susceptible to inflammatory damage. Our results showed that PFA supplemented to the diet improved growth performance, inhibited inflammatory response, and benefited liver protection, which provided a direction for the use of PFA in broiler production.

**Abstract:**

The current study aimed to explore the effects of supplementing paraformic acid (PFA) into broilers’ diet on growth performance, inflammatory responses, and liver protection. A total of 567 healthy one-day-old broilers were used in a 42-d study, and they were randomized into three groups. Broilers were fed a basal diet (CON group) or the basal diet supplemented with either 50 mg/kg aureomycin (AB group) or 1000 mg/kg PFA (PFA group). The results showed that the PFA and AB groups had a higher feed conversion rate than the CON group from day 21 to 42 (*p* < 0.05). Dietary PFA or aureomycin supplementation decreased serum levels of interleukin (IL)-1β, IL-6, IL-10, alanine transaminase, diamine oxidase, and D-lactate, and significantly increased serum concentrations of immunoglobulin (Ig) A, IgM, and complement C4 (*p* < 0.05). Moreover, dietary PFA or aureomycin supplementation decreased hepatic levels of caspase-1, NOD-like receptor family pyrin domain containing 3 (NLRP3), tumor necrosis factor-alpha, IL-6, and IL-18, as well as *NF-κB* mRNA expression (*p* < 0.05). Above all, PFA supplementation into the broilers’ diet improved growth performance, inhibited inflammatory responses, and benefited liver protection. The protective effects of PFA on the liver might be related to inhibition of caspase-1-induced pyroptosis via inactivating the NF-κB/NLRP3 inflammasome axis in broiler chickens.

## 1. Introduction

Livestock productivity and profits are dramatically increased by intensive poultry farming systems, but they also raise the risk of inflammatory injury in broiler chickens because of environmental aspects, pathogenic bacteria, and poor feed hygiene [1]. The liver, as a crucial metabolic organ, plays a momentous role in nutrients biosynthesis and metabolism, defense against bacterial invasion, and endotoxin clearance. Therefore, the liver is one of the organs that are most susceptible to inflammatory injury [2]. Liver injury would cause a reduction in the animals’ detoxification ability and the animals’ performance, thus imperiling the sustainable development of industrial livestock and poultry farming [3,4]. With the banning or limitation of antibiotic growth promoter (AGP) in animal husbandry, it is essential to look for new feed additives that can decrease liver inflammatory responses in broiler chickens. 

Formic acid (FA), a pure organic acid, has served as a feed preservative to guard feed against microbial and fungal contamination [5]. Studies indicated that supplementing FA into broiler chickens’ diet could elevate body weight (BW) gain and feed efficiency, which might be related to the reduced pH values of intestinal digesta which were beneficial in inhibiting harmful bacteria proliferation and increasing digestive enzymes’ activities and nutrients’ digestibility [6,7]. However, FA’s application in animal husbandry is constrained by its potent, pungent smell and corrosiveness to the gastrointestinal tract [8]. Therefore, FA derivatives are gradually applied to reduce the corrosive and pungent odor of FA [6,9]. Paraformic acid (PFA) is a hyperpolymer formed by polymerization of FA monomers. Our recent study indicated that 1000 mg/kg PFA supplementation into the broiler chickens’ diet could improve intestinal development and decrease intestinal inflammation via inactivation of the toll-like receptor 4 (TLR4)/nuclear factor-kappa B (NF-κB) pathway in broilers [10]. Moreover, Liu et al. [11] also demonstrated that the TLR4/NF-κB signaling pathway plays an important role in liver protection. However, there is no information available on the evaluation of effects of PFA supplementation into broiler chickens’ diet on the growth performance, inflammatory response, and liver protection in the scientific literature, and whether the TLR4/NF-κB signaling pathway is involved in liver protection by PFA needs to be studied.

Based on the above, this study aimed to explore the effects of supplementing PFA into broiler chickens’ diet on growth performance, inflammatory response, and liver protection based on the TLR4/NF-κB signaling pathway. As aureomycin was generally considered to be an effective AGP [11,12,13], and it was applied as a positive control in our study to highlight the possibility of the application of PFA in the poultry industry.

## 2. Materials and Methods

### 2.1. Animals and Diets

A total of 567 healthy 1-day-old Arbor Acres (AA) broilers with an initial average BW of 48.76 ± 1.42 g were used in a 42-d study. All broilers were randomized into 3 treatment groups (7 replicates per group and 27 broiler chickens per replicate) and housed in three-level cages in a light- and temperature-controlled room providing 24 h of constant illumination in the first three days and then 23 h of light and 1 h of dark until the end of the experiment. Broilers in three treatment groups were fed a basal diet (CON group) or fed the basal diet supplemented with either 50 mg/kg commercial aureomycin (AB group) [11,13] or 1000 mg/kg PFA (PFA group) [10]. The basal diets (Table 1) were provided to broilers in terms of a two-phase feeding program (0–21 d and 21–42 d) and formulated in compliance with the National Research Council (NRC, 1994). Paraformic acid used in this study was provided by Omega Nutrition Group (Spain) & Numega Nutrition Pte. Ltd. Throughout the experiment, feed and water were freely available to the broilers. On days 7 and 14 of the experiment, a Newcastle disease vaccine and an inactivated infectious bursal disease vaccine, respectively, were administrated. After being maintained at −35 °C for the first week, the room temperature was gradually reduced by 1 °C every two days until a final temperature of 21 °C was achieved. Body weights per replicate (cage) at 21 and 42 days were weighed after 12 h of fasting, and the average daily gain (ADG), average daily feed intake (ADFI), and feed-to-gain ratio (F/G) were calculated as previously described [11].

### 2.2. Sample Collection and Carcass Traits

At the end of the trial, a total of 21 broilers from different cages (seven per group), with a BW closest to the cage average, were selected for fasting blood samples and liver tissue samples collection. Blood samples (5 mL) were collected via the wing vein into the coagulation-promoting tubes and centrifuged for 15 min at 3500× *g* at 4 °C after standing at room temperature for 30 min. Serum samples were obtained, followed by being kept at −35 °C until further analysis. Then, the broilers were narcotized by CO_2_ asphyxiation after blood samples collection. The eviscerated yield percentage, abdominal fat percentage, breast muscle percentage, and thigh muscle percentage were calculated as previously described [14,15]. Moreover, about 2 g of liver samples were obtained from each broiler and stored at −80 °C for further analysis.

### 2.3. Determination of Serum Biochemical Indicators

The measurements of serum biochemical indicators, including total protein (TP), albumin (ALB), glucose (GLU), triglyceride (TG), high-density lipoprotein (HDL), low-density lipoprotein (LDL), and total cholesterol (TCHO) were performed with specific assays purchased from Nanjing Jiancheng Bioengineering Institute (Nanjing, China) as described in Liu et al. [11].

### 2.4. Determination of Serum Inflammatory Cytokines and Immunoglobulins Concentrations

The serum tumor necrosis factor-alpha (TNF-α), interleukin-1beta (IL-1β), interleukin-10 (IL-10), interleukin-6 (IL-6), immunoglobulin A (IgA), immunoglobulin G (IgG), and immunoglobulin M (IgM) were detected with chicken-specific assays (Jiangsu Meimian Industrial Co., Ltd., Zhangjiagang, China) as described previously [11].

### 2.5. Determination of Serum Diamine Oxidase (DAO) and D-Lactate Levels

Serum DAO activity and D-lactate concentration were analyzed by chicken-specific ELISA assays (Jiangsu Meimian Industrial Co., Ltd.) described in the Supplementary Methods.

### 2.6. Determination of Hepatic Injury Indexes, Inflammatory Cytokines, and Caspases Activities

Serum alanine transaminase (ALT) activity was determined using a commercially available kit according to the manufacturer’s instructions (Nanjing Jiancheng) on the Cobus-MiraPlus autoanalyzer. Serum complements (C3 and C4) concentrations and hepatic levels of 8-hydroxy-2’-deoxyguanosine (8-OHdG), heat shock protein 70 (HSP70), NOD-like receptor family pyrin domain containing 3 (NLRP3), TNF-α, IL-1β, IL-6, interleukin-18 (IL-18), caspase-1, and caspase-3 were examined with the assays (Jiangsu Meimian) on the basis of the protocol in the Supplementary Methods.

### 2.7. Determination of Hepatic Malondialdehyde (MDA) Concentration in Livers

Liver tissues were homogenized in 0.9% saline solution followed by centrifugation at 12,000× *g* for 15 min. The supernatants were obtained and prepared for MDA determination using a specific assay kit (Nanjing Jiancheng Bioengineering Institute, Nanjing, China) as described in Chen et al. [16].

### 2.8. Determination of Relative mRNA Expression in Livers

The detailed procedure of the relative mRNA expression determination in liver samples was described in the Supplementary Methods. Primer sequences, including *TLR4*, *NF-κB*, myeloid differentiation primary response 88 (*MyD88*), Bax, Bcl-2, sirtuin 1 (*Sirt1*), Heme oxygenase-1 (*HO-1*), necrosis-related factor 2 (*Nrf2*), superoxide dismutase-2 (*SOD2*), superoxide dismutase-1 (*SOD1*), NADPH quinone oxidoreductase 1 (*NQO1*), catalase (*CAT*), and glutathione peroxidase-1 (*GPX1*), used for real-time PCR are shown in Table 2. The β-actin gene was amplified in parallel as the internal control for gene normalization and quantification. The 2^−ΔΔ^ Ct method was used to calculate the relative target genes’ abundances in liver samples. All samples were measured in triplicate, and product sizes and quantities were determined by agarose gel electrophoresis.

### 2.9. Statistical Analyses

The average cage data were used to assess the effects on growth performance, and individual chicken data were used to access the effects on the other variables. Statistical analyses for all data were performed with one-way ANOVA of SAS (9.4 Inst. Inc., Cary, NC, USA). The POWER procedure of SAS was used to calculate the sample size [17]. Differences among the three groups were compared using Tukey’s multiple range tests. The Shapiro–Wilk W statistic was applied to check the normality of the data, and the data that were not normally distributed were transformed to achieve approximated normality. Values are presented as mean and standard error of mean (SEM) in tables, and values are expressed as mean ± standard error in Figures. Th statistical significance was set at *p* < 0.05, and a trend toward significance was considered at 0.05 ≤ *p* < 0.10.

## 3. Results

### 3.1. Effects of PFA Supplementation on Growth Performance

As shown in Table 3, relative to the broilers in CON group, the broilers in groups AB and PFA had significantly lower F/G from d 21 to 42 (*p* = 0.036) and tended to have decreased F/G from d 0 to 42 (*p* = 0.058). No significant difference was detected between the AB group and PFA group in terms of F/G throughout the experiment (*p* > 0.05). The body weight, ADFI, and ADG of the broilers did not show significant differences among all the groups during the experiment (*p* > 0.05).

### 3.2. Effects of PFA Supplementation on Carcass Traits

Indexes of the carcass traits of the broilers are displayed in Table 4. Broilers fed the AB diet had significantly higher breast muscle percentages than broilers fed the CON and PFA diets (*p* = 0.014). No statistically significant differences were detected in other indexes of the carcass traits among the three groups (*p* > 0.05).

### 3.3. Effects of PFA Supplementation on Serum Biochemical Indicators

There were no significant differences in serum concentrations of TP, ALB, GLU, TG, HDL, LDL, and TCHO among all treatments (*p* > 0.05, Table 5).

### 3.4. Effects of PFA Supplementation on Serum Inflammatory Cytokines and Immunoglobulins Concentrations

**Figure 1:**
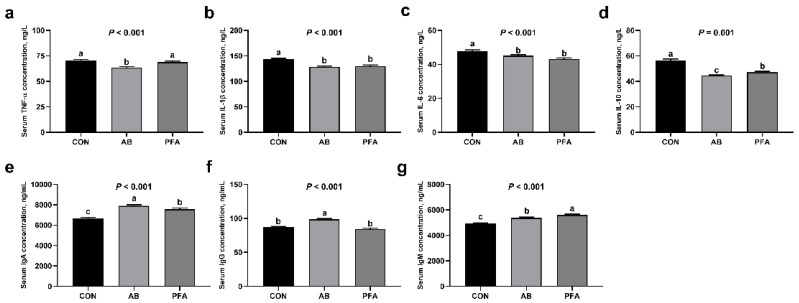
Effects of paraformic acid (PFA) supplementation on serum inflammatory cytokine and immunoglobulin concentrations in broiler chickens. (**a**) TNF-α, tumor necrosis factor-alpha; (**b**) IL-1β, interleukin-1 beta; (**c**) IL-6, interleukin-6; (**d**) IL-10, interleukin-10; (**e**) IgA, immunoglobulin A; (**f**) IgG, immunoglobulin G; (**g**) IgM, immunoglobulin M. CON, broiler chickens receiving basal diet; AB, broiler chickens receiving basal diet supplemented with 50 mg/kg aureomycin; PFA, broiler chickens receiving basal diet supplemented with 1000 mg/kg PFA. Values are mean ± standard error (*n* = 7). ^a,b,c^ Means with different superscripts differ (*p* < 0.05).

As presented in Figure 1, broilers in the AB group showed significantly reduced TNF-α concentrations and increased IgG concentrations in the serum compared with broilers in the CON and PFA groups (*p* < 0.05). Serum IL-6 and IL-1β concentrations in the AB group and PFA group were significantly lower than those in the CON group (*p* < 0.05), and serum IL-6 and IL-1β concentrations showed no significant differences between the AB group and the PFA group (*p* > 0.05). Serum IL-10 concentration was significantly lower in the AB and PFA groups than in the CON group (*p* < 0.05), and a significantly lower serum IL-10 concentration was observed in the AB group compared with the PFA group (*p* < 0.05). Groups AB and PFA showed markedly greater serum IgA concentrations than group CON (*p* < 0.05), while broilers in the AB group had significantly higher serum IgA concentrations than broilers in the PFA group (*p* < 0.05). Broilers in the PFA group displayed the highest serum IgM concentration, and the PFA group showed a significantly higher serum IgM concentration compared to the CON group and AB group (*p* < 0.05). Moreover, the serum IgM concentration in the AB group was significantly higher than that in the CON group (*p* < 0.05).

### 3.5. Effects of PFA Supplementation on Serum DAO and D-Lactate Levels

As shown in Table 6, the AB group and PFA group had significantly lower serum DAO activity and D-lactate concentrations than the CON group (*p* < 0.05).

### 3.6. Effects of PFA Supplementation on Hepatic Injury Indexes

**Figure 2:**
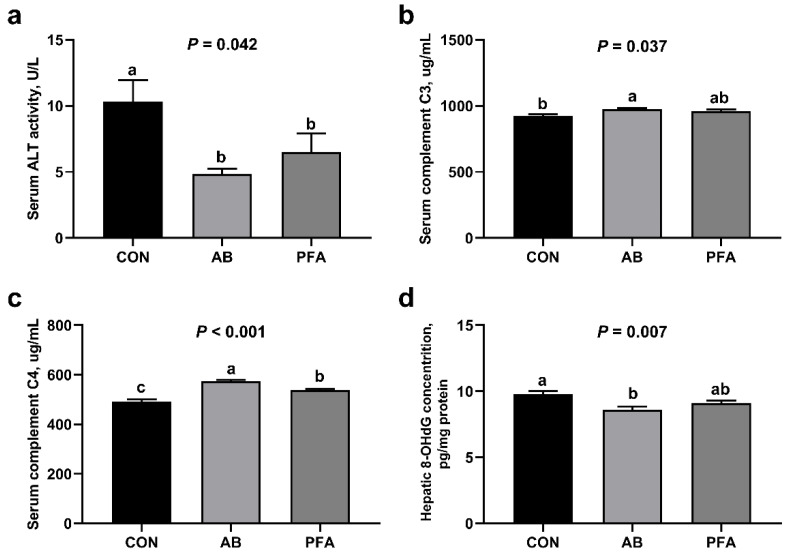
Effects of paraformic acid (PFA) supplementation on hepatic injury indexes in broiler chickens. (**a**) Alanine transaminase (ALT); (**b**) Complement C3; (**c**) Complement C4; (**d**) 8-OHdG, 8-hydroxy-2’-deoxyguanosine. CON, broiler chickens receiving basal diet; AB, broiler chickens receiving basal diet supplemented with 50 mg/kg aureomycin; PFA, broiler chickens receiving basal diet supplemented with 1000 mg/kg PFA. Values are mean ± standard error (*n* = 7). ^a,b,c^ Means with different superscripts differ (*p* < 0.05).

As shown in Figure 2, the serum ALT activity in the AB group and PFA group was significantly lower than that in the CON group (*p* < 0.05). Broilers in the AB group showed a significantly higher serum complement C3 concentration and a lower hepatic 8-OHdG concentration than broilers in the CON group (*p* < 0.05). Moreover, the complement C4 concentration in the serum of the AB group was significantly higher than that of the CON group and PFA group (*p* < 0.05), and the serum complement C4 concentration in the PFA group significantly increased compared with that in the CON group (*p* < 0.05). There were no significant differences in terms of ALT activity and complement C3 concentration in the serum and 8-OHdG concentration in the liver between the AB group and the PFA group (*p* > 0.05).

### 3.7. Effects of PFA Supplementation on Hepatic Apoptosis Indicators

**Figure 3:**
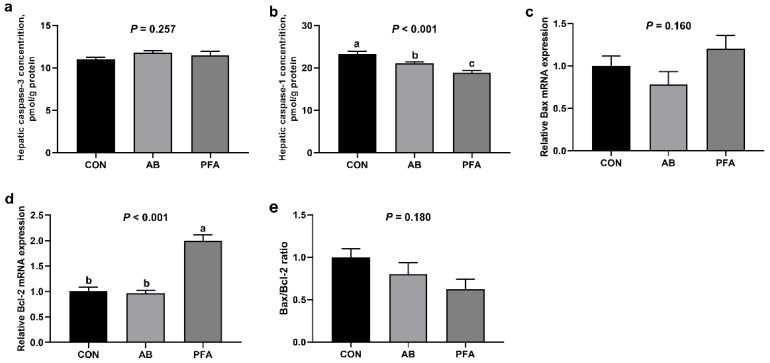
Effects of paraformic acid (PFA) supplementation on hepatic apoptosis indicators in broiler chickens. (**a**) Caspase-3; (**b**) Caspase-1; (**c**) *Bax*; (**d**) *Bcl-2*; (**e**) Bax/Bcl-2 ratio. CON, broiler chickens receiving basal diet; AB, broiler chickens receiving basal diet supplemented with 50 mg/kg aureomycin; PFA, broiler chickens receiving basal diet supplemented with 1000 mg/kg PFA. Values are mean ± standard error (*n* = 7). ^a,b,c^ Means with different superscripts differ (*p* < 0.05).

Hepatic apoptosis indicators of broilers are displayed in Figure 3. PFA Broilers showed significantly lower hepatic caspase-1 activity than the CON broilers and AB broilers (*p* < 0.05), and the hepatic caspase-1 activity of the AB group was markedly reduced compared with that of the CON group (*p* < 0.05). Moreover, the PFA group expressed significantly higher hepatic *Bcl-2* mRNA expression than the other groups (*p* < 0.05). However, the hepatic caspase-3 activity, *Bax* mRNA expression, and *Bax*/*Bcl-2* ratio were not significantly different among the three groups (*p* > 0.05).

### 3.8. Effects of PFA Supplementation on Hepatic Inflammatory Cytokines and Genes Expressions

**Figure 4:**
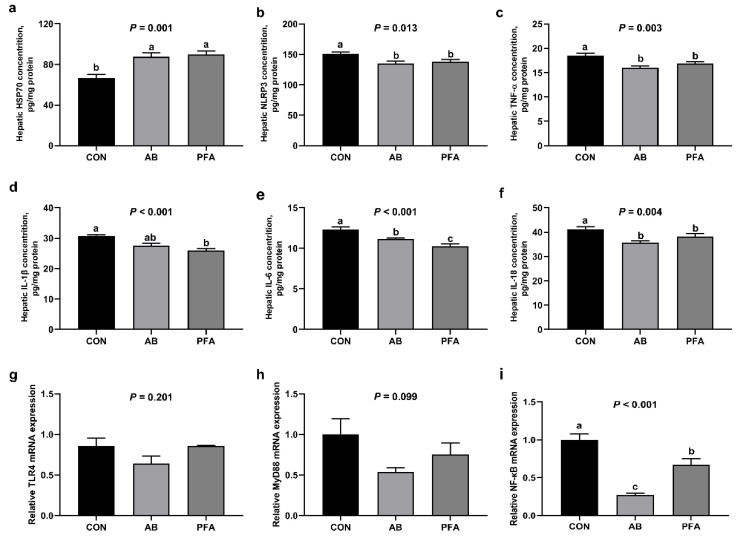
Effects of paraformic acid (PFA) supplementation on hepatic apoptosis indicators in broiler chickens. (**a**) HSP70, hepatic heat shock protein 70; (**b**) NLRP3, NOD-like receptor family pyrin domain containing 3; (**c**) TNF-α,tumor necrosis factor-alpha; (**d**) IL-1β, interleukin-1beta; (**e**) IL-6, interleukin-6; (**f**) IL-18, interleukin-1; (**g**) *TRL4,* toll-like receptor 4; (**h**) *MyD88,* myeloid differentiation primary response 88; (**i**) *NF-κB,* nuclear factor-kappa B. CON, broiler chickens receiving basal diet; AB, broiler chickens receiving basal diet supplemented with 50 mg/kg aureomycin; PFA, broiler chickens receiving basal diet supplemented with 1000 mg/kg PFA. Results are presented as mean ± SEM (*n* = 7). ^a,b,c^ Means with different superscripts differ (*p* < 0.05).

Hepatic inflammatory cytokines and genes expressions in broiler chickens are shown in Figure 4. Broilers in the AB group and PFA group showed significantly decreased NLRP3, TNF-α, and IL-18 concentrations and higher hepatic HSP70 levels compared with broilers in the CON group (*p* < 0.05). Compared to the CON group, dietary PFA supplementation significantly decreased hepatic IL-1β concentration (*p* < 0.05); supplementation of aureomycin and PFA significantly decreased hepatic IL-6 concentration (*p* < 0.05), and the hepatic IL-6 concentration in the PFA group was significantly lower than in the AB group (*p* < 0.05). Hepatic HSP70, NLRP3, TNF-α, IL-1β, and IL-18 concentrations showed no significant differences between the AB group and PFA group (*p* > 0.05). Moreover, compared with the CON group, supplementation of aureomycin or PFA significantly decreased hepatic *NF-κB* mRNA expression (*p* < 0.05), but the AB group showed a significant reduction in the hepatic *NF-κB* mRNA expression relative to the PFA group (*p* < 0.05). Neither *TRL4* nor *MyD88* expressions showed significant differences among all treatments (*p* > 0.05).

### 3.9. Effects of PFA Supplementation on Hepatic MDA Concentration and Antioxidant Genes Expressions

**Figure 5:**
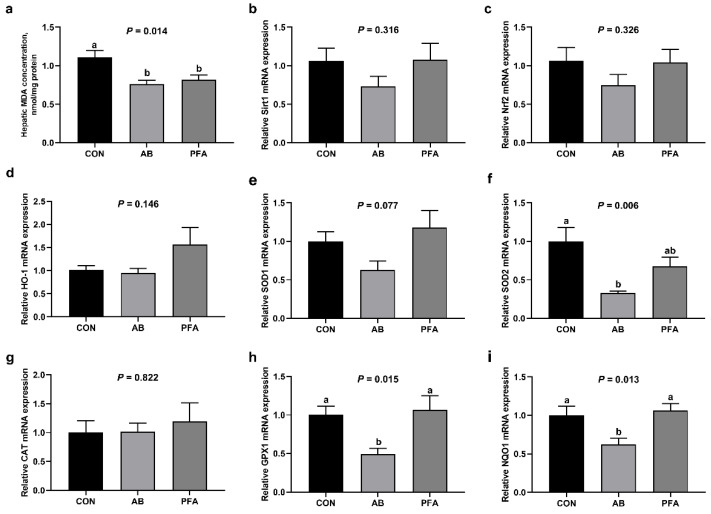
Effects of paraformic acid (PFA) supplementation on hepatic MDA concentration and antioxidant genes expressions in broiler chickens. (**a**) MDA, malondialdehyde; (**b**) *Sirt1,* sirtuin 1; (**c**) *Nrf2,* necrosis-related factor 2; (**d**) *HO-1,* heme oxygenase-1; (**e**) *SOD1,* superoxide dismutase-1; (**f**) *SOD2,* superoxide dismutase-2; (**g**) *CAT,* catalase; (**h**) *GPX1,* glutathione peroxidase-1; (**i**) *NQO1,* NADPH quinone oxidoreductase 1. CON, broiler chickens receiving basal diet; AB, broiler chickens receiving basal diet supplemented with 50 mg/kg aureomycin; PFA, broiler chickens receiving basal diet supplemented with 1000 mg/kg PFA. Results are presented as mean ± SEM (*n* = 7). ^a,b^ Means with different superscripts differ (*p* < 0.05).

As represented in Figure 5, relative to the CON group, the AB group and PFA group showed a significantly decreased hepatic MDA concentration (*p* < 0.05). Supplementation of aureomycin significantly decreased the hepatic *SOD2* mRNA expression (*p* < 0.05), and broilers fed the AB diet displayed significantly lower hepatic *GPX1* and *NQO1* expressions than broilers fed the CON diet and PFA diet (*p* < 0.05). Hepatic *SOD1* expression in the PFA group had a trend to be increased compared with that in the AB group (*p* < 0.10). There were no significant differences in *Sirt1*, *Nrf2*, *HO-1*, and *CAT* mRNA expressions among all groups (*p* > 0.05).

## 4. Discussion

The increasing spread of multi-resistant bacteria worldwide has posed a serious threat to global public health, which has led to growing discussion and concern about appropriate ways to use AGP in food animal production [18]. Formic acid is physiologically generated in animals’ metabolism and can also be obtained artificially by chemical synthesis [19]. Formic acid and its derivatives have been widely utilized in livestock feeds for decades because of its preservative and antimicrobial qualities [8,20]. Paraformic acid is a polymer formed by condensation of two FA molecules. Our recent study demonstrated that PFA supplementation was beneficial in improving intestinal development and changed the composition of cecal microbiota in broilers [10]. It has shown that aureomycin as AGP supplementation into the diet can promote broiler growth performance and carcass traits [21,22,23]. In the current study, decreased F/G and higher breast muscle percentage were also found in the broilers of the AB group compared with broilers of the CON group. Moreover, dietary PFA supplementation decreased F/G from d 21 to 42 and from d 0 to 42, suggesting that PFA had the potential to promote the growth of broiler chickens. 

Inflammation is a considerable factor resulting in the reduction of broiler growth performance [24]. Song et al. [25] demonstrated that supplementing aureomycin reduced serum IL-6, TNF-α, and IL-2 levels in broilers. In the present study, supplementation of 50 mg/kg aureomycin also decreased serum concentrations of proinflammatory cytokines, containing TNF-α, IL-1β, and IL-6, in broilers. Moreover, significantly decreased serum IL-1β and IL-6 concentrations were also observed in the PFA broilers in this study. Interleukin-1β is a master regulator of the physiological and pathological states and plays a vital role in inflammation-related diseases. Interleukin-1β has strong proinflammatory activity, triggering varieties of pro-inflammatory cytokines generation [26]. The pleiotropic inflammatory cytokine IL-6 plays a number of roles in inflammation and metabolic disease [27]. In addition, IL-1β could also induce IL-6 production [28]. Interestingly, supplementation of aureomycin or PFA decreased the serum IL-10 concentration of the broilers in the current experiment. The anti-inflammatory cytokine IL-10 is produced by CD (+) cells and plays a crucial role in maintaining tissue homeostasis in the presence of infection or inflammation [29]. Previous studies have demonstrated that an increased IL-6 concentration frequently induced an increase in IL-10 in the blood under chronic inflammatory conditions, which might be involved in the self-healing capacity of the body [30]. The results of the current study illustrated that dietary PFA was beneficial in reducing the body’s inflammatory response through inhibiting proinflammatory cytokines. On the other hand, dietary PFA supplementation increased the serum IgA and IgM concentrations, which was in accord with the effects of aureomycin addition. A previous study also found that aureomycin supplementation increased serum the IgM concentration of the broilers [22]. Immunoglobulins provide a first line of defense against extracellular infections for the host. Especially, as the first immunoglobulin expressed during B-cell development, IgM not only responds quickly to a variety of antigens, but also plays a vital role in immunoregulation [31]. Compared with IgM, IgA is a poor player in systemic immune response [32]. In this study, PFA broilers also showed a higher serum IgM concentration than AB broilers, suggesting that adding PFA to the diet enhanced the immune function of the broilers.

In addition, we found that supplementing PFA or aureomycin in the diets decreased the serum ALT concentration in the present study. Transaminase is the main enzyme involved in liver metabolism and will be released into the circulation once the hepatocytes are damaged, leading to a dramatically increased concentration in the blood [33]. The liver of poultry is more and more easily damaged because of the improper use of chemical drugs, molds, and environmental factors in the extensive poultry farming industry [1]. The results of ALT suggested decreased hepatocyte damage in the broilers in the PFA and AB group. Moreover, we examined the serum concentrations of complements C3 and C4, as well as the hepatic concentration of 8-OHdG. Proteins of the complement systems are produced by hepatocytes. Hepatic dysfunction is often accompanied by reduced serum complement C3 and C4, so complement C3 and C4 can be available markers to assess liver function and hepatocellular damage [34]. DNA damage often produced 8-OHdG in the nucleotide pool during DNA replication, so 8-OHdG is regarded as the most significant marker of DNA damage [35]. A previous study has shown that aureomycin supplementation decreased the 8-OHdG concentration in the serum and jejunum of the broilers [36]. The results in this study demonstrated that dietary PFA and aureomycin both played protective roles in the liver.

To further investigate the underlying mechanism of the protective effects of dietary PFA supplementation on the livers of broiler chickens, hepatic caspases activities and relative mRNA expressions of pro-apoptotic Bax and anti-apoptotic Bcl-2 were examined. In the present study, supplementation with 1000 mg/kg PFA increased the *Bcl-2* mRNA expression and decreased caspase-1 activity. The Bcl-2 family play a key role in the control of apoptosis, and Bcl-2 functions upstream as a caspase activation inhibitor [37]. The cysteine protease caspase-1 is an evolutionarily conserved inflammatory mediator, and it proteolytically cleaves and matures IL-1β and IL-18 in answer to NLRP3 inflammasome activation [38]. Subsequently, the caspase-1 activates pyroptosis that is a type of inflammation-induced cell death triggered by the cytosolic danger signals or pathogens [39]. Consistently, decreased hepatic NLRP3 inflammasome and IL-1β and IL-18 concentrations were found in the PFA group in the present study. Studies in vitro showed that NF-κB signaling is an essential prerequisite for an efficient activation of NLRP3 inflammasome in primary hepatocytes [40]. In this study, we also found that supplementation of PFA reduced *NF-κB* mRNA expression as well as TNF-α and IL-6 concentrations in the liver. It was reported that NF-κB signaling was a key inducer of inflammation, and blockade of NF-κB activation restrained TNF-α and IL-6 generation [41]. Moreover, 1000 mg/kg PFA addition elevated hepatic HSP70 level in this study. Heat shock protein 70 s are crucial parts of the cellular network of folding catalysts and molecular chaperones. Martine et al. [42] indicated that HSP70, serving as an NLRP3 inflammasome inhibitor, played protective roles against hepatic inflammatory cytokines in chickens and mice [43,44,45]. Decreased inflammatory cytokines concentrations and NF-κB expression, as well as caspase-1 activity, were also found in the broilers fed the aureomycin-supplemented diet in this study. Therefore, our study demonstrated that dietary PFA might be beneficial to liver protection through decreasing caspase-1-induced pyroptosis via inactivating the NF-κB/NLRP3 inflammasome axis. 

A persistently active inflammatory response can result in an overproduction of reactive oxygen species, which can harm critical cellular components by oxidizing proteins, modifying DNA histones, and lipids [46]. In our study, broilers in the AB group and PFA group exhibited decreased hepatic MDA concentrations compared with broilers in the CON group. Malondialdehyde is a secondary product of lipid oxidation and closely related to cell damage, so it is widely considered as an indicator to evaluate the extent of lipid peroxidation [47]. The results in this study might demonstrate that PFA or aureomycin supplementation reduced hepatic oxidative damage. However, PFA or aureomycin supplementation did not increase the expression of hepatic antioxidation-related genes, which might suggest that PFA or aureomycin benefited liver health primarily by reducing the hepatic inflammatory response in broiler chickens. The down-regulated mRNA expressions of *SOD2*, *GPX1*, and *NQO1* in the AB group compared with the CON group might be associated with the decreased inflammation-induced oxidative stress [36].

In the current study, dietary PFA or aureomycin supplementation reduced serum DAO and D-lactate concentrations. The serum DAO and D-lactate levels are usually considered markers for assessing the extent of intestinal mucosal damage [48]. Damaged intestinal mucosal could lead to increased intestinal permeability and the release of intracellular enzyme DAO and bacteria-produced D-lactate from the intestine into the blood [49]. The decreased serum levels of DAO and D-lactate demonstrated that dietary PFA or aureomycin supplementation was helped to maintain intestinal mucosal integrity, which was in accordance with Li et al. [10]. Previous studies have also shown that supplementation of FA and its derivatives, as well as aureomycin, could improve intestinal development and maintain the normal intestinal barrier structure of broilers [6,50]. The liver is situated between the absorptive surfaces of the gastrointestinal tract, and its blood supply mostly comes from the gut via the hepatic portal vein. The damaged intestinal mucosa would cause the translocation of bacterial products to the liver, resulting in liver function impairment [51]. Therefore, these results suggested that the protective effect of PFA or aureomycin supplementation on liver health might be partly attributed to decreased intestinal injury.

## 5. Conclusions

In conclusion, dietary PFA addition could promote growth performance, inhibit inflammatory responses and benefit liver protection. The protective effects of PFA on the liver might be related to the suppression of caspase-1-induced pyroptosis through blockage of the NF-κB/NLRP3 inflammasome axis in the broiler chickens. Our study provided a direction for the use of PFA in broiler production.

## Figures and Tables

**Table 1:** Ingredients composition and nutrient levels of basal diets (as-fed basis).

Items	Phases	
	0–21 d	21–42 d
Ingredients, %		
Corn	55.91	55.91
Soybean meal, 44% CP	13.78	10.18
Wheat bran	11.98	12.98
Corn starch residue	7.99	9.98
Corn gluten meal	3.99	3.99
Extruded soybean	1.50	2.10
Limestone	1.70	1.70
Calcium monophosphate	1.10	1.10
L-Lysine HCl	1.00	1.00
DL-Methionine	0.20	0.20
L-Threonine	0.10	0.10
Sodium chloride	0.40	0.40
Choline	0.10	0.10
Phytase	0.10	0.10
Complex enzyme	0.02	0.02
Trace mineral premix ^1^	0.10	0.10
Vitamin premix ^2^	0.02	0.02
Antioxidant	0.02	0.02
Total	100	100
Calculated analysis, %		
Metabolizable energy, MJ/kg	12.33	12.50
Crude protein	19.47	17.93
Crude fat	3.45	3.74
Calcium, %	0.94	0.87
Available phosphorus, %	0.35	0.33
Lysine, %	1.15	1.00
Methionine, %	0.50	0.40

^1^ Provided per kilogram of complete basal diet: 10 mg of Cu as CuSO_4_, 100 mg of Fe as FeSO_4_, 1.1 mg of I as Ca(IO_3_)_2_, 65 mg of Zn as ZnSO_4_, 100 mg of Mn as MnSO_4_ and 0.3 mg of Se as Na_2_SeO_3_. ^2^ Provided per kilogram of complete basal diet: vitamin A 10,000 IU, vitamin D_3_ 3000 IU, vitamin E 30 IU, vitamin K_3_ 1.3 mg, vitamin B_1_ 2.2 mg, vitamin B_2_ 8 mg, vitamin B_3_ 8 mg, vitamin B_6_ 4 mg, vitamin B_12_ 0.025 mg, biotin 0.2 mg, niacin 40 mg, folic acid 1 mg and D-calcium pantothenate 10 mg.

**Table 2:** Primer sequences used for quantitative real-time PCR.

Genes	Gene Bank No.	Primer Sequences ^a^ (5’-3’)	Size, bp
*β-actin*	NM_205518.1	F: TTGGTTTGTCAAGCAAGCGG	100
		R: CCCCCACATACTGGCACTTT	
*TLR4*	NM_001030693.1	F: AGGCACCTGAGCTTTTCCTC	96
		R: TACCAACGTGAGGTTGAGCC	
*MyD88*	XM_046910878.1	F: TGATGCCTTCATCTGCTACTG	174
		R: TCCCTCCGACACCTTCTTTCTA	
*NF-κB*	NM_001396038.1	F: CAGCCCATCTATGACAACCG	152
		R: TCAGCCCAGAAACGAACCTC	
*Bax*	XM_422067	F: GGTGACAGGGATCGTCACAG	108
		R: TAGGCCAGGAACAGGGTGAAG	
*Blc-2*	NM_205339.2	F: GCTGCTTTACTCTTGGGGGT	128
		R: CTTCAGCACTATCTCGCGGT	
*Sirt1*	XM_046920057.1	F: GATCAGCAAAAGGCTGGATGGT	143
		R: ACGAGCCGCTTTCGCTACTAC	
*Nrf2*	XM_015289381.2	F: CCCGCACCATGGAGATCGAG	180
		R: GGAGCTGCTCTTGTCTTTCCT	
*HO-1*	NM_205344.1	F: GTCGTTGGCAAGAAGCATCC	106
		R: GGGCCTTTTGGGCGATTTTC	
*SOD1*	NM_205064.1	F: GGCAATGTGACTGCAAAGGG	133
		R: CCCCTCTACCCAGGTCATCA	
*SOD2*	NM_204211.2	F: CTTGGTCGCAAGGCAGAAG	120
		R: ACGTAGGTGGCGTGGTGTT	
*CAT*	NM_001031215.1	F: GGTTCGGTGGGGTTGTCTTT	211
		R: CACCAGTGGTCAAGGCATCT	
*GPX1*	HM590226	F: AACCAATTCGGGCACCAG	122
		R: CCGTTCACCTCGCACTTCTC	
*NQO1*	NM_001277619.1	F: AACCTCTTTCAACCACGCCA	113
		R: GTGAGAGCACGGCATTGAAC	

^a^ F, forward; R, reverse.

**Table 3:** Effects of paraformic acid (PFA) supplementation on growth performance of broilers.

Items	Treatments ^a^			SEM	*p* Value
	CON	AB	PFA		
Body weight, g					
d 0	47.60	47.63	47.60	0.59	0.999
d 21	775.70	765.39	789.37	8.60	0.544
d 42	2171.33	2234.93	2252.67	33.81	0.604
Average daily feed intake, g/d					
d 0–21	57.32	57.33	58.37	0.60	0.723
d 21–42	142.12	137.91	140.26	2.84	0.835
d 0–42	99.72	97.62	99.32	1.56	0.846
Average daily gain, g/d					
d 0–21	34.67	34.18	35.32	0.41	0.549
d 21–42	66.46	69.98	69.68	1.50	0.590
d 0–42	50.57	52.08	52.50	0.81	0.610
Feed-to-gain ratio					
d 0–21	1.66	1.68	1.65	0.01	0.558
d 21–42	2.14 ^a^	1.97 ^b^	2.01 ^b^	0.02	0.036
d 0–42	1.90	1.83	1.83	0.01	0.058

Values are presented as mean and standard error of mean (SEM). ^a,b^ Means with different superscripts within a row differ (*p* < 0.05). ^a^ CON, broiler chickens receiving basal diet; AB, broiler chickens receiving basal diet supplemented with 50 mg/kg aureomycin; PFA, broiler chickens receiving basal diet supplemented with 1000 mg/kg PFA. *n* = 7.

**Table 4:** Effects of paraformic acid (PFA) supplementation on carcass traits in broiler chickens.

Items, %	Treatments ^a^			SEM	*p* Value
	CON	AB	PFA		
Eviscerated yield percentage	73.68	71.61	72.46	1.44	0.399
Abdominal fat percentage	1.26	1.18	1.36	0.07	0.221
Breast muscle percentage	23.02 ^b^	26.89 ^a^	22.88 ^b^	0.92	0.014
Thigh muscle percentage	16.71	18.06	19.24	0.39	0.310

Values are presented as mean and standard error of mean (SEM). ^a,b^ Means with different superscripts within a row differ (*p* < 0.05). ^a^ CON, broiler chickens receiving basal diet; AB, broiler chickens receiving basal diet supplemented with 50 mg/kg aureomycin; PFA, broiler chickens receiving basal diet supplemented with 1000 mg/kg PFA. *n* = 7.

**Table 5:** Effects of paraformic acid (PFA) supplementation on serum biochemical indicators in broiler chickens.

Items	Treatments ^a^			SEM	*p* Value
	CON	AB	PFA		
TP, g/L	28.55	26.42	31.22	1.63	0.499
ALB, g/L	7.55	6.63	7.57	0.42	0.594
HDL, mmol/L	1.50	1.41	1.67	0.06	0.205
LDL, mmol/L	0.46	0.48	0.61	0.05	0.430
TCHO, mmol/L	2.81	2.49	3.08	0.12	0.158
TG, mmol/L	0.56	0.40	0.47	0.05	0.439
GLU, mmol/L	11.12	10.52	11.60	0.49	0.673

Values are presented as mean and standard error of mean (SEM). ^a^ CON, broiler chickens receiving basal diet; AB, broiler chickens receiving basal diet supplemented with 50 mg/kg aureomycin; PFA, broiler chickens receiving basal diet supplemented with 1000 mg/kg PFA. *n* = 7.

**Table 6:** Effects of paraformic acid (PFA) supplementation on serum diamine oxidase and D-lactate levels in broiler chickens.

Items	Treatments ^a^			SEM	*p* Values
	CON	AB	PFA		
Diamine oxidase, pg/mL	144.28 ^a^	126.78 ^b^	128.57 ^b^	1.04	<0.001
D-lactate, μg/L	487.66 ^a^	452.83 ^b^	460.83 ^b^	4.02	<0.001

Values are presented as mean and standard error of mean (SEM). ^a,b^ Means with different superscripts within a row differ (*p* < 0.05). ^a^ CON, broiler chickens receiving basal diet; AB, broiler chickens receiving basal diet supplemented with 50 mg/kg aureomycin; PFA, broiler chickens receiving basal diet supplemented with 1000 mg/kg PFA. *n* = 7.

## Data Availability

Not applicable.

## Supplementary Materials

The following supporting information can be downloaded from: https://www.mdpi.com/article/10.3390/ani12202825/s1. Supplementary Methods: Detailed procedures of ELISA analysis; Determination of relative mRNA expression in livers.
