# Supplementary material for: Supplementation of Paraformic Acid as a Substitute for Antibiotics in the Diet Improves Growth Performance and Liver Health in Broiler Chickens
Source: Animals (Basel). 2022 Oct 18;12(20):2825. doi: 10.3390/ani12202825 (PMC9597723; doi:10.3390/ani12202825)
Supplement: Supplementary file 1 [file animals-12-02825-s001.zip › animals-1912098-supplementary.pdf]

SUPPLEMENTARY METHODS

Detailed Procedures of ELISA Analysis

Liver tissues were homogenized in 0.9% saline solution followed by centrifugation at 12,000 × g for 15 min. The serum or prepared supernatants of tissue homogenate (50 μL) and diluted standard solutions were added to the corresponding microplates. After reacting for 30 min at 37 °C, the microplates were washed five times and 50 μl of the HRP-Conjugated Reagent was added to each well. Then the microplates were cultured for 30 min at 37 °C and washed five times again. Chromogenic procedure was performed with two kinds of chromogenic agents for 10 min at 37 °C followed by Stop Buffer addition. Finally, the absorbance of each well was read within 15 min, and the concentrations of supplements C3, supplement C4, IgA, IgG, IgM, 8-OHdG, HSP70, NLRP3, TNF-α, IL-1β, IL-6, IL-10, IL-18, caspase-1, and caspase-3 were calculated using the standard curve made with standard solutions.

Determination of Relative mRNA Expression in Livers

Frozen liver tissue samples (50-100 mg) were ground to a powder in a mortar to which liquid nitrogen was continually added. Total RNA was extracted using TRIzol reagent (Invitrogen, Carlsbad, CA, USA). RNA quality was analyzed by 1.0% agarose gel electrophoresis at 80 V for 25 min at a low temperature. Gels were observed under ultraviolet light for clear bands with no smearing. The absorbance of RNA solutions was measured at wavelengths of 260 nm and 280 nm using a Beckman DU-800 scanning spectrophotometer (Beckman Coulter Inc., Brea, CA, USA). RNA concentrations were confirmed using a Beckman DU-800 nucleic-acid/protein analyzer

(Beckman Coulter Inc.). cDNA was then synthesized using a commercial reverse transcription (RT) kit (TaKaRa Biotechnology, Tokyo, Japan) according to the manufacturer’s instructions and stored at −20°C for relative quantification by polymerase chain reaction (PCR) using a CFX-96 real-time PCR detection system (Bio-Rad, Hercules, CA, USA). The cDNA was amplified using an ABI 7900HT instrument (Applied Biosystems, Foster City, CA, USA). The mixture (10 mL) contained 5 mL SYBR Green Supermix (TaKaRa Biotechnology), 1 mL cDNA, 0.4 mL each primer (10 mM), 0.2 mL ROX Reference Dye and 3 mL ddH_2O. The cycling conditions were as follows: pre-denaturation at 95°C for 30 s, followed by 40 cycles of denaturation at 95°C for 5 s and annealing at 60°C for 34 s. To confirm the specificity of each product, melting curve analysis (50°C increased to 95°C at a rate of 0.1°C/sec with continuous fluorescence measurements) was performed.
